# Leptin, Leptin Receptor, KHDRBS1 (KH RNA Binding Domain Containing, Signal Transduction Associated 1), and Adiponectin in Bone Metastasis from Breast Carcinoma: An Immunohistochemical Study

**DOI:** 10.3390/biomedicines8110510

**Published:** 2020-11-17

**Authors:** Paola Maroni, Alessandro Luzzati, Giuseppe Perrucchini, Luca Cannavò, Paola Bendinelli

**Affiliations:** 1 IRCCS Istituto Ortopedico Galeazzi, Laboratory of Experimental Biochemistry & Molecular Biology, Via R. Galeazzi 4, 20161 Milano, Italy; 2 IRCCS Istituto Ortopedico Galeazzi, Oncological Orthopedics and Reconstructive Surgery, Via R. Galeazzi 4, 20161 Milano, Italy; alessandro.luzzati@grupposandonato.it (A.L.); lucacannav@gmail.com (L.C.); 3 Fondazione Istituto Giglio di Cefalù, Via Pietrapollastra Contrada Pisciotto, 90015 Cefalù (Pa), Italy; giuseppe.perrucchini@hsrgiglio.it; 4 Dipartimento di Scienze Biomediche per la Salute, Università degli Studi di Milano, Via L. Mangiagalli 31, 20133 Milano, Italy

**Keywords:** bone metastasis, KH RNA binding domain containing, signal transduction associated 1 (KHDRBS1), leptin, leptin receptor (LEPR), adiponectin

## Abstract

Breast cancer patients are at a high risk of complications from bone metastasis. Molecular characterization of bone metastases is essential for the discovery of new therapeutic targets. Here, we investigated the expression and the intracellular distribution of KH RNA binding domain containing, signal transduction associated 1 (KHDRBS1), leptin, leptin receptor (LEPR), and adiponectin in bone metastasis from breast carcinoma and looked for correlations between the data. The expression of these proteins is known in breast carcinoma, but it has not been investigated in bone metastatic tissue to date. Immunohistochemical analysis was carried out on bone metastasis specimens, then semiquantitative evaluation of the results and the Pearson test were performed to determine eventual correlations. KHDRBS1 expression was significantly higher in the nuclei than in the cytosol of metastatic cells; LEPR was prevalently observed in the cytosol and the nuclei; leptin and adiponectin were found in metastatic cells and stromal cells; the strongest positive correlation was between nuclear KHDRBS1 and nuclear LEPR expression. Taken together, our findings support the importance of the leptin/LEPR/KHDRBS1 axis and of adiponectin in the progression of bone metastasis and suggest their potential application in pharmacological interventions.

## 1. Introduction

Breast cancer is the second leading cause of death among women worldwide [1]. Although mortality rates have declined, the incidence after menopause continues to rise. Therapeutic failure is largely due to the heterogeneity of breast cancer subtypes and clinical features, as well as the spread and development of secondary growth in bone and lung and associated complications [2]. Bone metastases often occur in breast cancer [3]. Bone is a special organ: mineral content, matrix composition, extreme rigidity, highly hypoxic environment, acidic pH, and high concentrations of extracellular calcium create a fertile ground for neoplastic cells to grow. The hallmarks of bone metastases are skeletal fractures, nerve compression, and pain—so-called skeletal-related events (SREs)—that worsen the quality of life and compromise survival [4].

It is necessary to deepen the knowledge of the molecular features of the metastases to understand the molecular mechanisms that regulate the progression of the lesions in the bone tissue to find targets that can offer the opportunity to treat metastatic disease and improve the quality of life of patients. One of the major problems for this type of investigation is the impossibility to recover a sufficiently large number of samples, since biopsies of bone metastases can be obtained only from patients undergoing surgery for bone consolidation or pathological fractures.

KH RNA binding domain containing, signal transduction associated 1 (KHDRBS1) also known as Src-associated substrate during mitosis of 68 KDa (Sam68), is a protein of the signal transduction and activation of RNA metabolism (STAR) family of RNA-binding proteins (RBPs) that link signalling pathways to RNA processing [5,6]. The functions of KHDRBS1 have been recently correlated with tumour progression in breast cancer [7]. KHDRBS1 is upregulated in breast cancer cells and breast tumours [8,9,10]; it can induce epithelial-to-mesenchymal transition (EMT) and migration in breast cancer cells. KHDRBS1 upregulation and cytosolic localization are associated with poor prognosis [11] in renal cell carcinoma and breast cancer [8], while in other types of cancers nuclear localization and high KHDRBS1 expression are associated with tumour progression and poor prognosis [12]. To date, KHDRBS1 has not been studied in bone metastatic tissue.

Stimuli from membrane receptors involve KHDRBS1 in signalling events, modulate its affinity for RNA, and regulate its subcellular localization. KHDRBS1 is also involved in the signalling pathway of leptin receptor (LEPR) in breast cancer cells [13] as a transducer that mediates the effects of the hormone on cell proliferation and growth. In response to leptin, KHDRBS1 also modulates cell responsiveness to leptin through regulation of the alternative splicing of mRNA for LEPR [14].

Leptin, an adipokine secreted by adipose tissue, promotes tumour invasiveness and growth of breast cancer cells through autocrine and paracrine loops [15]. Leptin forms a link between obesity and cancer: obesity is associated with the onset of breast cancer and the development of drug resistance [15]; however, little is known about how obesity contributes to the metastasis.

The pattern of tissue distribution of KHDRBS1, leptin, and LEPR in bone metastases from breast cancer is unknown. With the present study, we investigated the expression and the intracellular distribution of KHDRBS1, leptin, and LEPR in bone metastasis from breast carcinoma and looked for eventual correlations between them. We also investigated adiponectin, another adipokine implicated in the crosstalk between adipose tissue and breast carcinoma [16,17]. Our hypothesis was that the leptin/KHDRBS1 axis, if functional in the metastatic cell, could number among the factors contributing to colonization of bone tissue.

## 2. Experimental Section

### 2.1. Tissue Collection and Preparation

Tissue samples were collected from patients undergoing surgical removal of metastatic tissue and bone stabilization at IRCCS Istituto Ortopedico Galeazzi, Milano. All subjects gave their informed consent for participation in the study. The study was conducted in accordance with the tenets of the Declaration of Helsinki of 1975. The procedure for the use of waste material for research purposes was approved by the Institutional Review Board of IRCCS Istituto Ortopedico Galeazzi (PQ 7.5.125, version 4, 22.01.2015). All samples were anonymized, and no information or images that could lead to identification of a study participant might ever occur.

### 2.2. Immunohistochemistry Analysis (IHC)

Bone samples were fixed in 10% neutral buffered formalin, decalcified in Mielodec (Bio-Optica, Milan, Italy) and embedded in paraffin. One serial section (4 μm) of each specimen from 22 patients was stained with haematoxylin and eosin following standardized methods to determine the presence of bone metastatic cells. The slides were de-waxed in xylene and re-hydrated in a graded series of ethanol. After antigen retrieval (95 °C in a water bath for 20 min at pH 6 in antigen-unmasking solution, Vector Laboratories, Burlingame, CA, USA), the sections were treated for 10 min with 0.1% (*v*/*v*) H_2_O_2_ to block endogenous peroxidase activity, and then blocked with normal serum. Immunostaining was performed overnight at 4 °C on bone specimen slices with anti-leptin (1:200, Proteintech, Manchester, UK), anti-LEPR (1:100, Proteintech), anti-KHDRBS1/Sam68 (1:200, EPR3232 Abcam, Cambridge, UK), or anti-adiponectin (1:200, 19F1 Abcam) antibody, using a streptavidin-biotin system (ABC staining system, Santa Cruz Biotechnology, Dallas TX, USA) and 3,3′-diaminobenzidine substrate, and counterstaining with Meyer’s haematoxylin [18]. Negative-control sections underwent the same staining procedure without the primary antibody. The chosen antibodies were validated for immunohistochemistry analysis (IHC) by the manufacturers and were tested in pilot experiments by titration. Immunoreactivity in at least 10 different section fields was measured by light microscopy (CKX41 Olympus, Olympus Co., Tokyo, Japan), and the mean percentage of tumour cells displaying positive staining was scored.

### 2.3. Statistical Analysis

Student’s t tests and analysis of variance (ANOVA) were used to test the immunohistochemical expression of each protein. Pearson’s correlation analysis was used to test pairwise correlations between the biomarkers (GraphPad, Prism 5, GraphPad Software Inc., San Diego, CA, USA). Statistical significance was set at *p* < 0.05.

## 3. Results

### 3.1. Characterization of Bone Metastasis Samples

Bone metastases were collected from 22 patients during surgery for bone consolidation or for pathological skeletal fractures. For each sample, the data of biomarkers routinely used in clinical practice (Ki67, oestrogen receptor (ER), progesterone receptor (PR), human epidermal growth factor receptor 2 (HER2)), as assessed by histological analyses, were retrieved from the medical records (Table 1). The proliferation rate as indicated by Ki67 was elevated in at least 68% of the samples; 95% of the samples were ER+, and 45% were PR+; 14% of the samples were positive for HER2.

**Figure 1:**
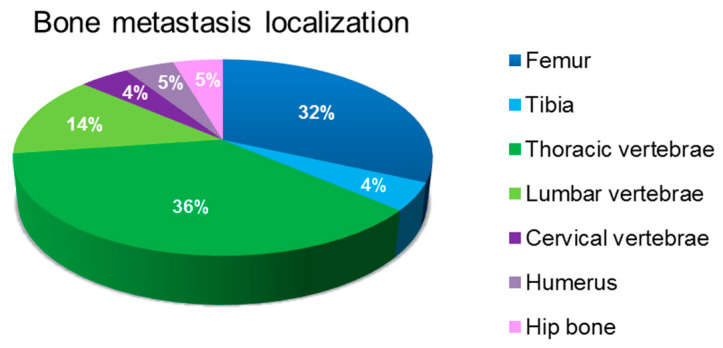
Anatomical site of bone metastasis.

Figure 1 presents the anatomical site of bone metastasis. The cohort considered in the present study reflects the frequency of anatomical sites of bone involvement.

**Figure 2:**
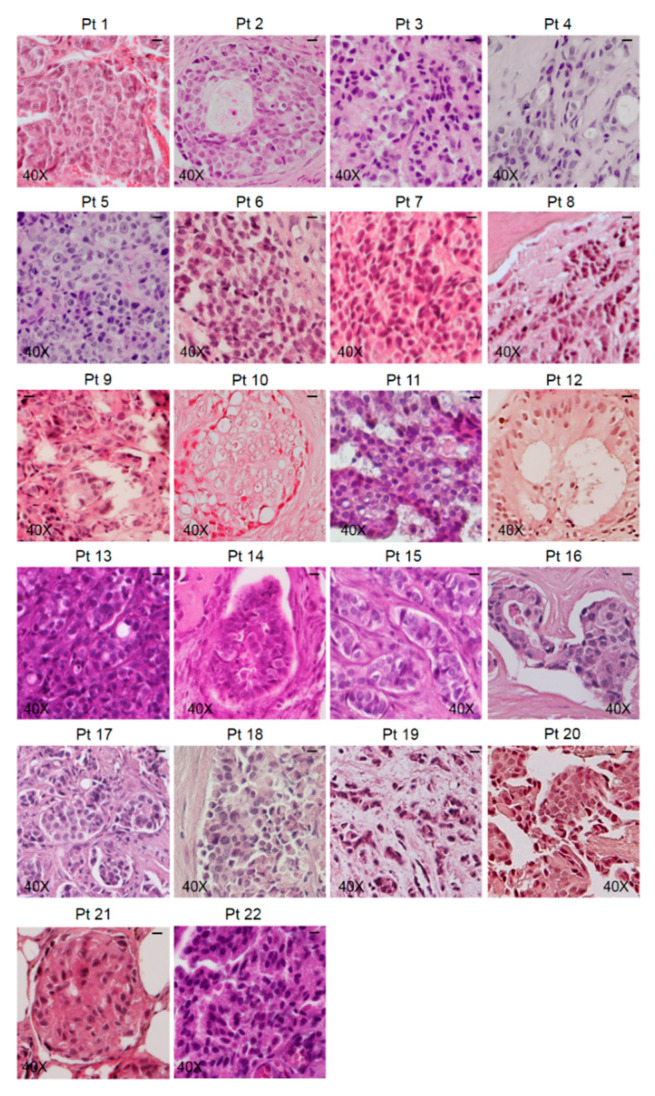
Standard haematoxylin and eosin (H&E) staining was performed on the specimens obtained from each patient, and representative images are shown. The scale bar represents 50 μm.

Bone metastasis samples underwent haematoxylin and eosin (H&E) staining to determine the suitability of the specimens for the presence of metastatic cells (Figure 2).

More than one sample was obtained from each patient during surgery, but only the specimens that showed the presence of metastatic cells underwent IHC analysis. Representative images of the samples from each patient are shown in Figure 2.

### 3.2. Pattern of Expression of KHDRBS1, Leptin, Leptin Receptor (LEPR), and Adiponectin in Human Bone Metastatic Tissue from Breast Cancer

**Figure 3:**
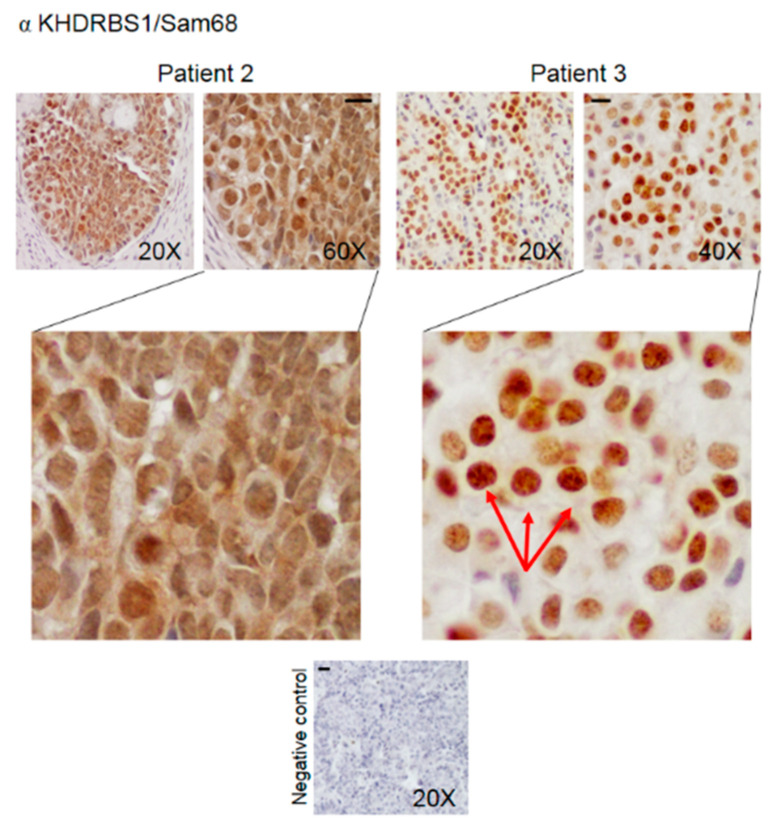
Representative IHC staining images (two patients) of human bone metastasis from breast cancer stained with anti-KHDRBS1 antibody. The arrows indicate KHDRBS1 associated with nuclear bodies. The scale bar represents 50 μm.

**Figure 4:**
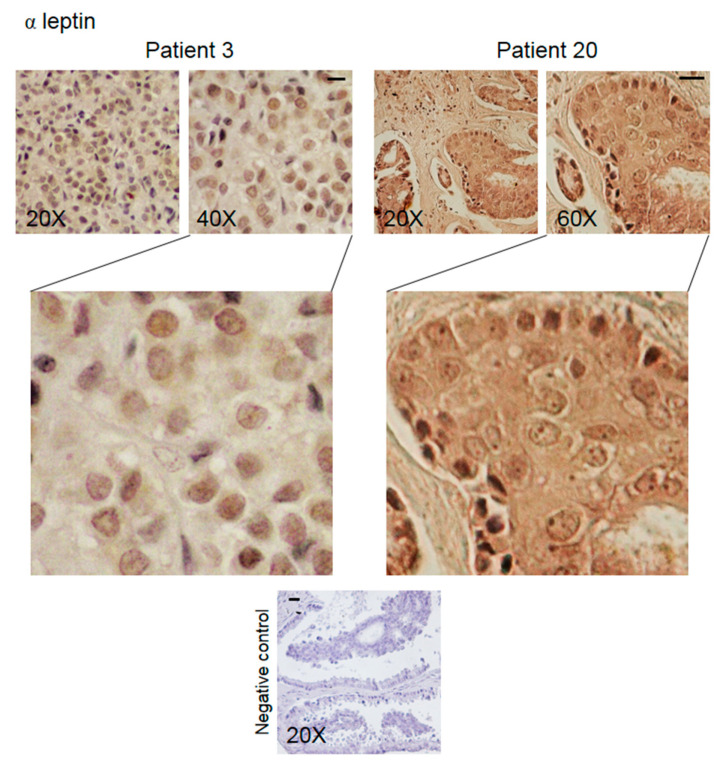
Representative IHC staining images (two patients) of human bone metastasis from breast cancer stained with anti-leptin antibody. The scale bar represents 50 μm.

**Figure 5:**
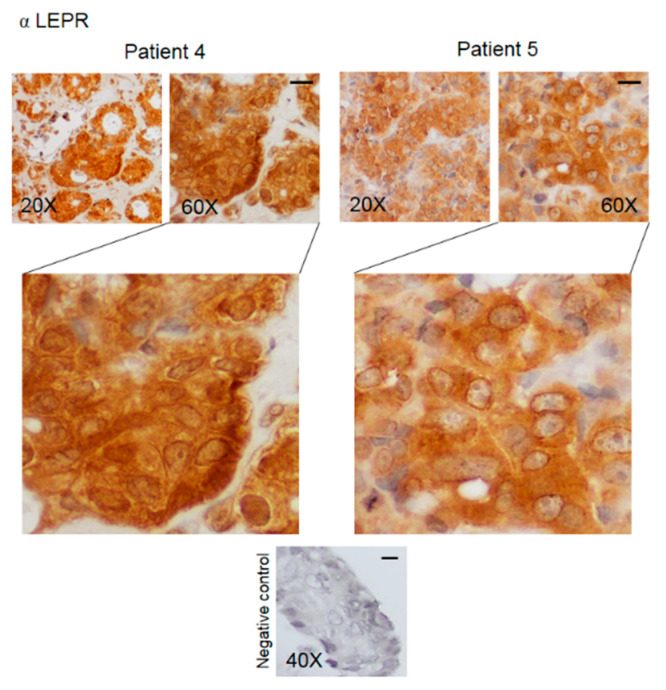
Representative IHC staining images (two patients) of human bone metastasis from breast cancer stained with anti-LEPR antibody. The scale bar represents 50 μm.

**Figure 6:**
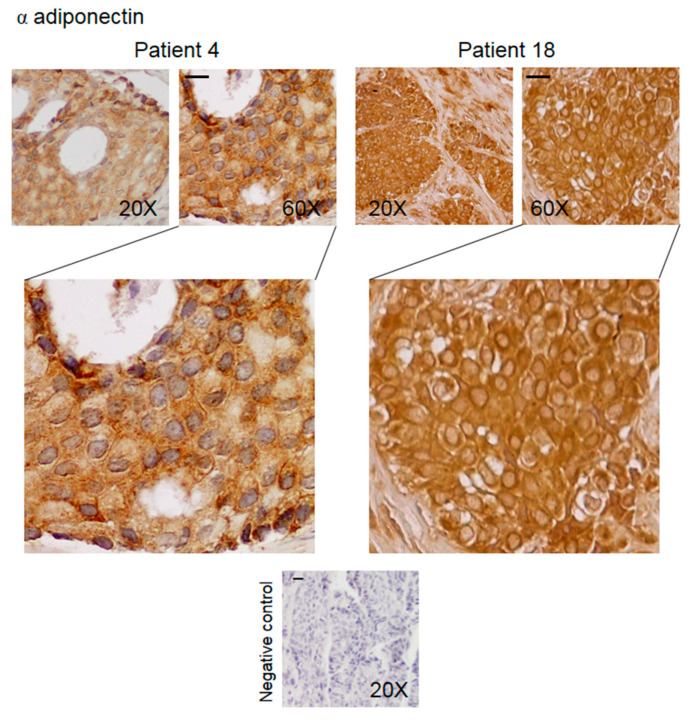
Representative IHC images (two patients) of human bone metastasis from breast cancer stained with anti-adiponectin antibody. The scale bar represents 50 μm.

Immunohistochemical analysis was performed to determine the expression and the intracellular distribution of KHDRBS1, leptin, LEPR, and adiponectin in bone metastatic tissue. The immunoreactivity of the studied parameters has been examined for staining intensity, and the presence of positive cells was quantified using a semiquantitative method. Immunoreactivity intensity was scored as 0 (no staining), >0/≤1 (low staining), >1/≤2 (moderate staining), and >2 (high staining). The samples were grouped into four groups (Table 2); representative images of IHC of proteins from the samples from two patients are shown in Figure 3, Figure 4, Figure 5 and Figure 6. The results for the other 20 patients are presented in the Supplementary Material (Figures S1–S4).

All the biomarkers were expressed in at least 73% of the metastatic tissue samples. KHDRBS1 expression was significantly higher in the nuclei than in the cytosol (** *p* = 0.002); high levels of nuclear KHDRBS1 were found in 68% of the samples, 72% were PR negative. Low or moderate levels of KHDRBS1 were detected in the cytosol (Table 2, Figure 3 and Figure S1). The nuclear signal appeared concentrated in KHDRBS1 nuclear bodies (SNBs, Figure 3 red arrows) consistent with the capacity of the protein to associate ribonucleoprotein complexes [19].

Leptin expression was detected in metastatic cells (86% of the samples) and in some cells in the microenvironment (27% of the samples); the level of hormone expression was moderate (50% of the samples) (Table 2, Figure 4 and Figure S2).

LEPR staining was observed in the cytosol and the nuclei of cancer cells and was significantly higher in the cytosol (§ *p*= 0.018); LEPR levels in the cytosol were high in 68% of the samples (Table 2, Figure 5 and Figure S3).

Adiponectin immunoreactivity was moderate/high in nearly all (86%) of the samples. Staining for adiponectin was observed in the metastatic cells and in the stromal cells in 45% of the samples (Table 2, Figure 6 and Figure S4).

Negative controls did not show specific signals in all the experiments.

Taken together, all the biomarkers were present in the metastatic tissue samples, suggesting a possible role for the leptin/LEPR/KHDRBS1 axis and adiponectin in the colonization/spread of metastatic cells.

### 3.3. Correlations between the Examined Parameters

We searched for significant correlations to determine the meaning of the observed patterns of protein expression.

**Figure 7:**
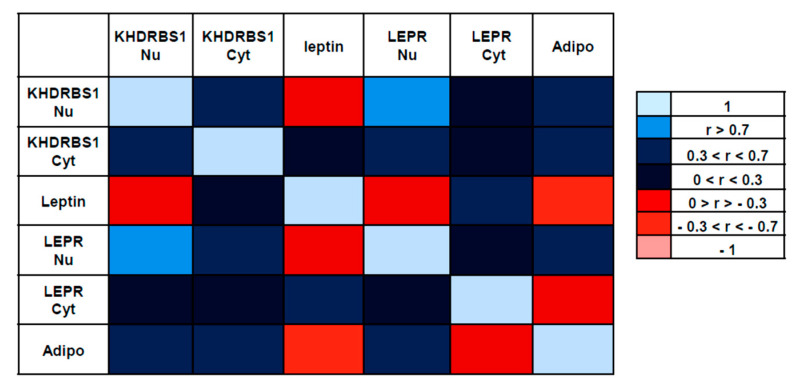
Pairwise Pearson correlation matrix for IHC expression of KHDRBS1, leptin, LEPR, and adiponectin. The blue boxes represent variables that have a positive relationship, while the red boxes represent variables that have a negative relationship. The lighter the box, the closer the correlation is to one, negative or positive. KHDRBS1 Nu: nuclear KHDRBS1; KHDRBS1 Cyt, cytosolic KHDRBS1; LEPR Nu, nuclear leptin receptor; LEPR Cyt, cytosolic leptin receptor; Adipo, adiponectin.

The strongest positive correlation was observed between nuclear KHDRBS1 and nuclear LEPR (r = 0.7290; *p* = 0.0001); there was also a moderate correlation between nuclear KHDRBS1 and adiponectin (r = 0.5191; *p* = 0.0067). Cytosolic KHDRBS1 showed a moderate positive correlation with adiponectin (r = 0.3707; *p* = 0.0447), and a moderate positive correlation was also observed between leptin and its cytosolic receptor (r = 0.5313; *p* = 0.0055) (Figure 7).

A moderate negative correlation was observed between leptin and adiponectin (r = −0.3539; *p* = 0.053); adiponectin is known to exert potent anti-tumour activities, whereas leptin exhibits pro-tumorigenic properties in breast cancer [20].

We also tested correlations between the biomarkers and the receptorial asset (ER, PR, HER2) and the proliferation index (Ki67). There was a moderate negative correlation between nuclear KHDRBS1 and PR expression (r = −0.3758; *p* = 0.0424) and a moderate positive correlation between cytosolic KHDRBS1 and Ki67 expression (r = 0.5520; *p* = 0.0088).

## 4. Discussion

Bone metastases represent a relevant clinical problem due to the impairment of the quality of life for patients. Even if some neoplastic diseases show a tropism for the skeleton, such as breast and prostate carcinomas and multiple myeloma, bone metastases also represent a relevant problem in advanced cancers. The introduction of biological therapies prolonged the overall survival of cancer patients and this is correlated to an increase in the risk of developing bone metastases in patients with solid tumours. For example, it has been reported that in patients affected by renal cell carcinoma [21] or by gastric cancer [22], the increased survival, due to the introduction of new therapies, is correlated to an augmented possibility to develop bone metastasis. Biological characterization of bone metastases can help elucidate the molecular mechanisms underlying lesion progression. Here, we considered bone metastasis derived from breast carcinoma to investigate the expression in bone metastasis of biomarkers KHDRBS1, leptin, LEPR, and adiponectin. The pattern of expression of such parameters is known in breast carcinoma [23,24,25,26,27], but it has never been studied in metastasis derived from breast cancer. A comparison with healthy tissue was not performed, because it was considered not pertinent to the present study, which aimed to investigate the molecular characterization of metastatic cells. Leptin, a hormone that regulates food intake and energy balance and is overexpressed in obesity [28], has been implicated as a link between breast cancer and obesity, which represents a risk factor for breast cancer [17]. In this context, it is important to understand how obesity affects tumour progression and metastasis development. The leptin/LEPR/KHDRBS1 axis, whose components have been studied in breast cancer [13], may also be expressed in metastatic tissue and contribute to colonization. The present results demonstrate the expression of these biomarkers in metastatic cells.

A particularly interesting finding is the marked nuclear localization of KHDRBS1: in breast cancer, its overexpression in the cytosol correlates with poor prognosis [8]. Our data suggest that KHDRBS1 may have different functions in the two phases of tumour growth. In the cytoplasm, KHDRBS1 interacts with Src, growth factor receptor-bound protein 2 (Grb2), and Grb2-related adaptor protein (Grap) and stimulates oncogenic pathways, including the epidermal growth factor pathway, extracellular signal-regulated kinase (ERK), and protein kinase B (Akt) pathways [8,29,30,31]. Cytoplasmic and nuclear localization of KHDRBS1 may contribute to neoplastic transformation or tumour progression through diverse molecular mechanisms in different cancer types or cellular contexts. Our results indicate a prevalent nuclear localization of KHDRBS1, mostly concentrated in the nuclear bodies (SNBs). This observation is consistent with what is known about the subcellular localization of the protein. SNBs, which are often observed in transformed cells such as breast cancer cells [32], contain splicing regulators, signalling components, and nucleic acids; SNBs assemble and disassemble depending on the transcriptional state of the cells, and they are probably involved in the transport of mRNA through the nucleus [19,33]. In the nuclei, KHDRBS1 is also involved in the regulation of gene transcription: it binds the transcriptional cofactor CREB binding protein (CBP) [34], which can lead to the regulation of proliferation.

Here, we show that, in bone metastatic tissue, KHDRBS1 is also present in the cytoplasm where it forms a link that guides extracellular signals towards RNA processing and/or regulation of transcription [8]. A role for KHDRBS1 in cancer progression has been suggested by Valacca et al., who observed that KHDRBS1 regulates the transformation of epithelial cancers by up-regulating the expression of the proto-oncogene SF2/ASF, which is important for the decision of EMT vs. mesenchymal-to-epithelial transition (MET) program and a crucial event in the formation of metastasis [35]. KHDRBS1 also promotes the progression of human breast cancer by up-regulating the activation of a surface antigen (EphA3), probably leading to the development of metastasis [36]. Moreover, the positive correlation between KHDRBS1 expression and the expression of matrix-metalloproteinase-9 (MMMP-9) in breast cancer tissue [36] supports the role of KHDRBS1 in the promotion of breast cancer metastasis. Our findings provide evidence for involvement of KHDRBS1 in the colonization of bone tissue and extend its role in neoplastic progression. The correlation between KHDRBS1 expression and the expression of LEPR strengthens the idea that KHDRBS1 is implicated downstream of the LEPR in metastatic cells, as has been reported in breast cancer cells [13].

The distribution of LEPRs is similar to that of related receptors for prolactin and erythropoietin, which are also found in intracellular pools [37,38]. LEPR is present on the cell surface for a short period: after binding with circulating leptin, the leptin/LEPR complex is internalized through clathrin-mediated endocytosis, then degraded by a cellular lysosomal system [39,40]. We observed that the LEPR is distributed in nuclear and cytosolic fractions in metastatic cells. Al-Shibli et al. reported that the LEPR is highly concentrated in the nucleus of breast cancer cells, indicating that the nucleus is the principal site of hormone action [27]. The researchers went on to explain that LEPR overexpression has a fundamental role in breast carcinogenesis, since it enables cancer cells to internalize circulating leptin. The presence of the hormone receptor in bone metastasis here reported demonstrates that metastatic cells are also responsive to leptin, suggesting that obesity can contribute to metastasis development.

We observed that bone metastatic tissue stained for leptin in cancer cells and stromal cells: leptin is one of the many microenvironmental factors that act on metastatic cells, as occurs in the mammary glands [41]. Furthermore, leptin expression in metastatic cells may indicate that the carcinoma cells maintain their ability to produce the hormone in the new growth site or that they have internalized the hormone from the circulation or the microenvironment. Therefore, leptin can contribute to the progression of bone lesions via autocrine and/or paracrine mechanisms. Of note, leptin is able to make cells responsive to itself, through the positive regulation of LEPR mRNA operated by KHDRBS1 [14]. In this way, KHDRBS1 may play a role in amplifying the response of bone metastasis to leptin, which would underscore the relevance of the leptin/LEPR system in tumour progression.

We also investigated adiponectin, an adipokine that is released in breast adipose tissue and appears to counteract leptin in the progression of breast cancer. In vivo and in vitro studies have reported that, in ER negative breast cancer cells, adiponectin negatively influences cell proliferation, invasion, and migration, and that it induces cell growth arrest and apoptosis [42,43,44,45,46]. In ER-positive breast cancer cells, however, adiponectin increases cell proliferation [47,48], indicating a role of ER status and the effect exerted by adiponectin. Crosstalk between leptin and adiponectin may exist, by which the two biomarkers influence each other in regulating mitogenic and/or apoptotic pathways [26]. Our findings show that all the samples expressed adiponectin in the cancer cells and that adiponectin was expressed in the stromal cells of 45% of patients. Like leptin, it may act as an autocrine and/or paracrine hormone and contribute to cancer cell colonization. Further studies are in progress to elucidate the role of the crosstalk between these adipokines in bone metastasis.

In this study, we evaluated whether the leptin/LEPR/KHDRBS1 axis, reported in the cellular model as relevant to tumour progression, could be expressed in bone metastatic tissue in vivo. Its presence in bone metastatic tissue underlines its involvement in the development and spread of metastases in the skeleton. Blocking bone metastases when they are in an early stage, also acting on leptin/LEPR/KHDRBS1 axis, could be a way to reduce the negative and destructive impact that inevitably leads to a poor prognosis for patients. However, the study is descriptive and the function of the leptin/LEPR/KHDRBS1 axis will need to be investigated and confirmed by in vitro studies. Another limitation of this study is the impossibility to recover the pair matched breast carcinomas to compare the pattern of distribution and the levels of expression of the considered parameters, but our study was stimulated by the knowledge of the expression of the parameters under examination in breast cancer. Despite these limitations, our results represent a good background and deserve to be considered in further research.

## 5. Conclusions

Bone metastases from breast cancer express components of the leptin/LEPR/KHDRBS1 axis in an expression pattern that partly follows that of the primary tumour but also exhibits peculiarities. The marked expression of KHDRBS1 in the nucleus and its presence in the cytoplasm of metastatic cells suggest that KHDRBS1 can perform a wider range of functions in metastasis than in carcinoma. These observations underscore the importance of KHDRBS1 in regulating tumour progression. Moreover, the responsiveness of metastatic cells to leptin, as well as the presence of adiponectin in metastatic tissue, supports the importance of the microenvironment in directing metastatic colonization. The present findings advance our knowledge of the biology of bone metastases from breast cancer and could be useful in future studies on therapeutic intervention.

## Figures and Tables

**Table 1:** Patient characteristics: receptorial asset (ER, PR, HER2), and Ki67 status.

Characteristic	No.	%
**Age at surgery (years)**		
<50	4	18
>50 <70	12	55
>70	6	27
**Ki67 status**		
high (>15%)	15	68
low (<15%)	3	14
n.e.	4	16
**ER status**		
ER+	21	95
ER−	1	5
**PR status**		
PR+	10	45
PR−	12	55
**HER2 status**		
HER2+ (>3+)	3	14
HER2−	17	77
n.e.	2	9

ER, oestrogen receptor; PR, progesterone receptor; HER2, human epidermal growth factor receptor 2; n.e., not evaluable.

**Table 2:** Semiquantitative results of immunohistochemistry analysis (IHC) analysis: number of patients for each group.

Staining Intensity	KHDRBS1 Nuclei	KHDRBS1 Cytosol	Leptin	LEPR Nuclei	LEPR Cytosol	Adiponectin
Negative	4	6	3	1	1	/
Low	1	5	5	4	1	3
Moderate	2	10	11	10	5	11
High	15 (68%)	1	3	7	15	8
Mean score (*n* = 22)	2.24 ± 0.29 **	1.09 ± 0.18	1.55 ± 0.19	1.84 ± 0.19	2.5 ± 0.19 ^§^	2.11 ± 0.14

** *p* = 0.002 vs. KHDRBS1 in the cytosol; ^§^ *p* = 0.018 vs. leptin receptor (LEPR) in the nuclei.

## Supplementary Materials

The following material is available online at https://www.mdpi.com/2227-9059/8/11/510/s1, Figure S1: IHC analysis of the expression of KHDRBS1 in human bone metastasis from breast cancer; Figure S2: IHC analysis of the expression of leptin in human bone metastasis from breast cancer; Figure S3: IHC analysis of the expression of KHDRBS1 in human bone metastasis from breast cancer; Figure S4: IHC analysis of the expression of adiponectin in human bone metastasis from breast cancer.
