# Supplementary figures and images for: Leptin, Leptin Receptor, KHDRBS1 (KH RNA Binding Domain Containing, Signal Transduction Associated 1), and Adiponectin in Bone Metastasis from Breast Carcinoma: An Immunohistochemical Study
Source: Biomedicines. 2020 Nov 17;8(11):510. doi: 10.3390/biomedicines8110510 (PMC7698510; doi:10.3390/biomedicines8110510)

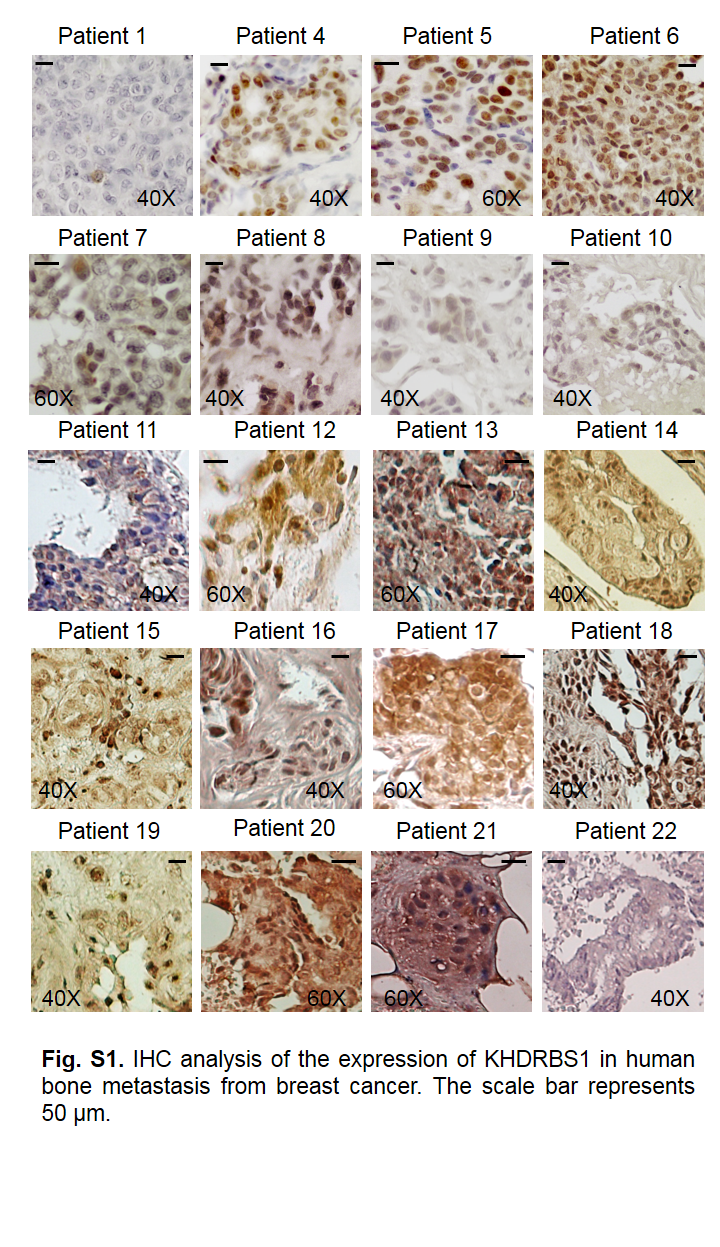

Supplement: Supplementary file 1 [file biomedicines-08-00510-s001.zip › Maroni et al. Fig S1.tif]

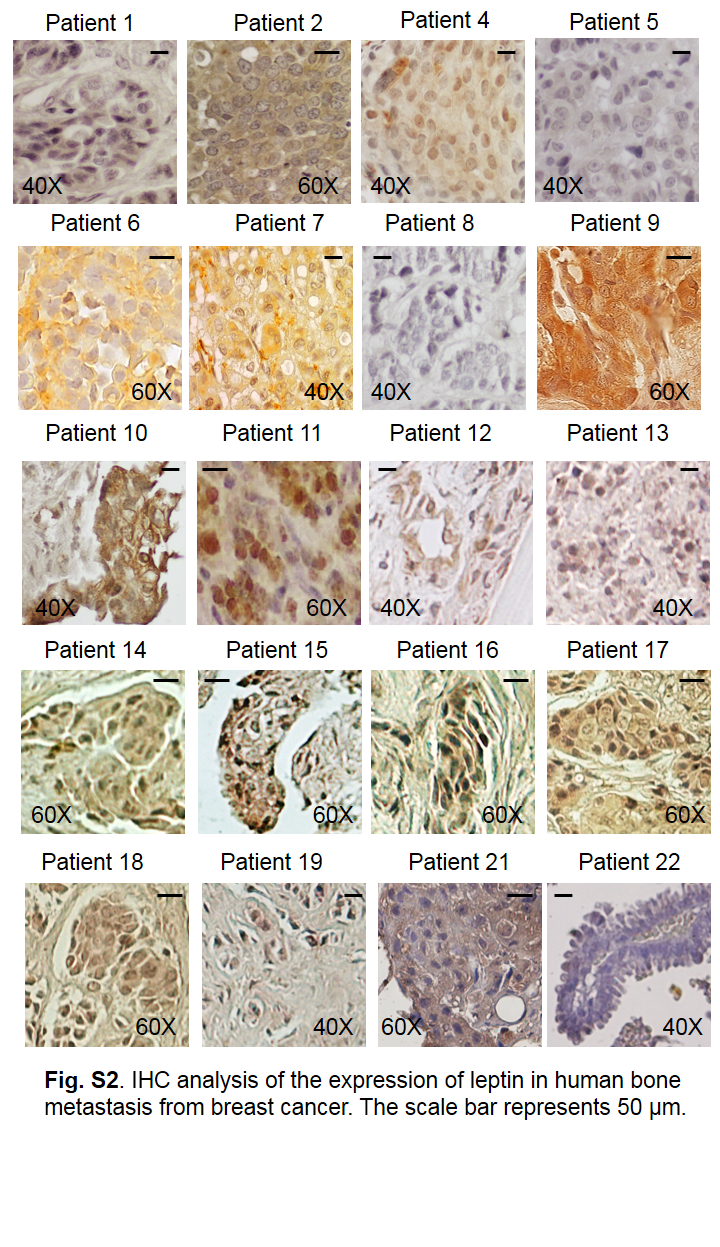

Supplement: Supplementary file 1 [file biomedicines-08-00510-s001.zip › Maroni et al. Fig S2.tif]

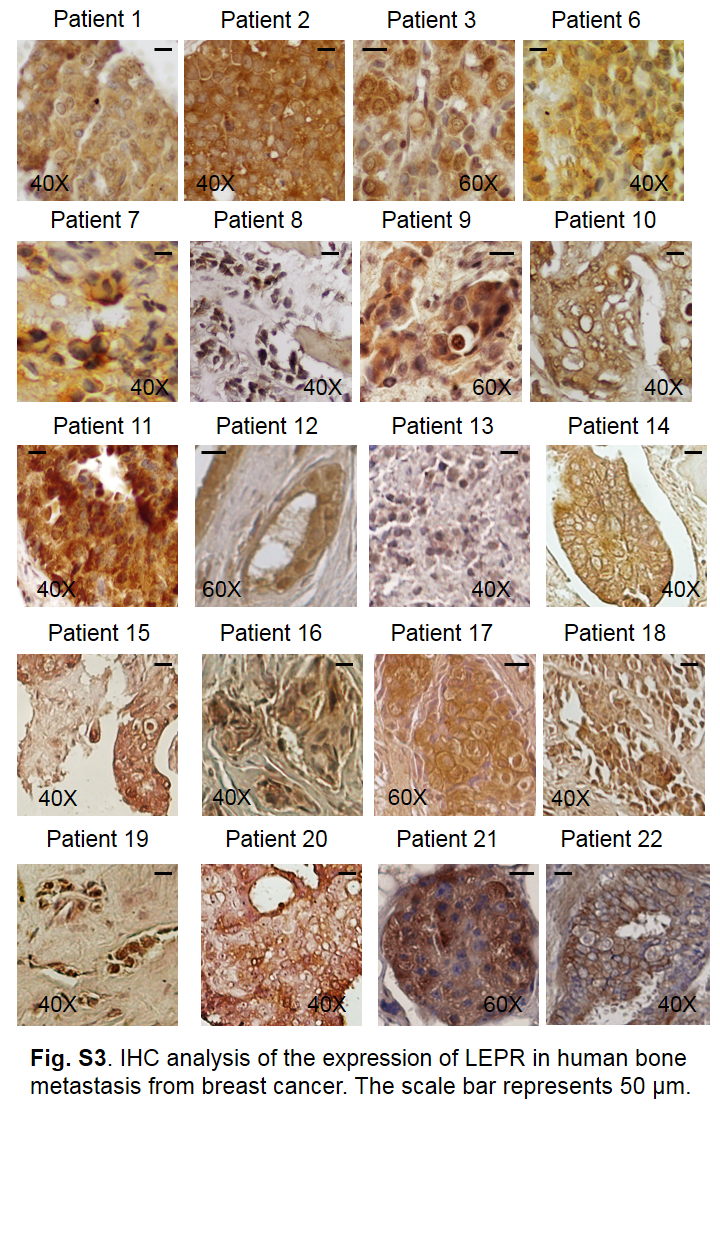

Supplement: Supplementary file 1 [file biomedicines-08-00510-s001.zip › Maroni et al. Fig S3.tif]

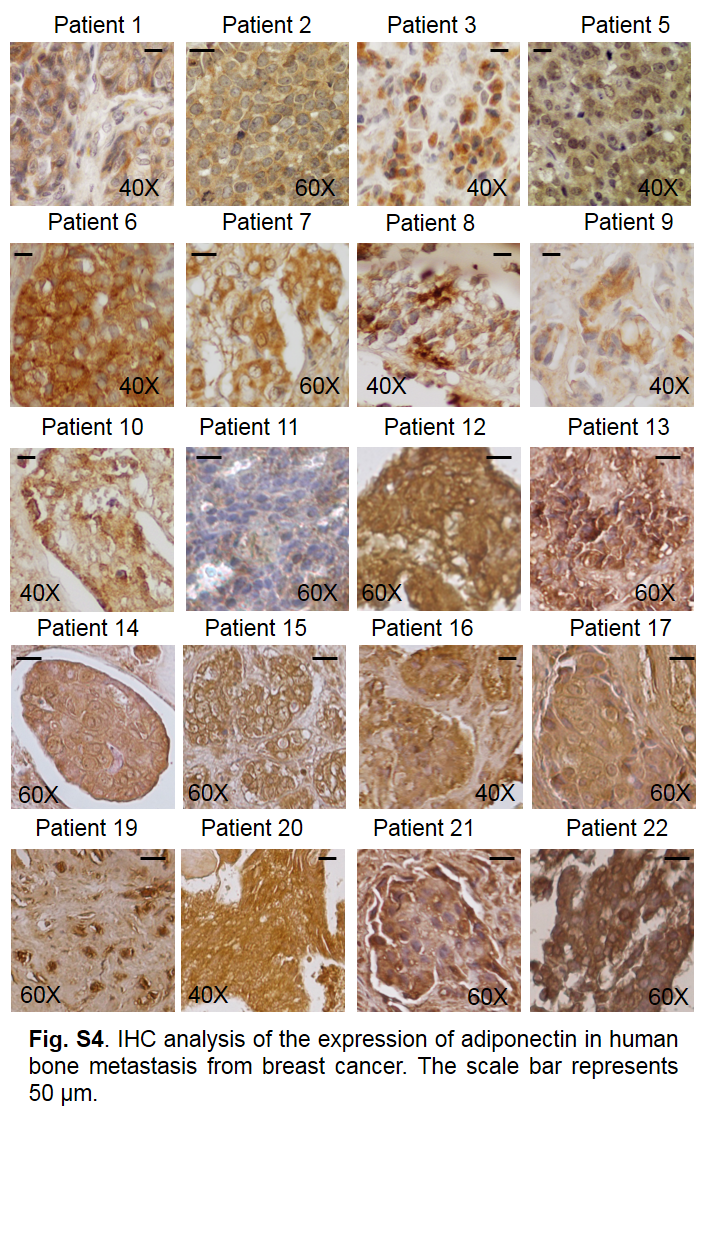

Supplement: Supplementary file 1 [file biomedicines-08-00510-s001.zip › Maroni et al. Fig S4.tif]
